# Semi-Annual Meeting of the Æsculapian Society

**Published:** 1856-07

**Authors:** D. W. Stormant, Wm. M. Chambers

**Affiliations:** President; Sec. P. T.


*Semi-Annual Meeting of the Æsculapian Society, held at York, Ill., May 28th*, 1856.

The Æsculapian Society of Illinois, met in semi-annual session at York, this day, and at 11 o'clock, (the President, Dr. S. York, of Paris, and the Vice-President, Dr. D. W. Stormant, of Grandview, being both absent,) on motion of Dr. Chambers, of Charleston, Dr. Mitchell, of Darwin, was called to the chair, when Dr. Hamilton (Secretary) pleading sickness, Dr. Chambers, at his request, read the proceedings of the preceding meeting, after which they were, on motion, corrected, so as to divide the special from the standing committees; and that it be understood that special committees are to make their reports the meeting next after their appointment, whether it be at the annual or semi-annual sessions, and that standing committees make their reports at the annual meetings only, (except the Committees on Arrangements and Publication;) and, further, that the case reported by Dr. Banks, at the last meeting, should have been noticed in the last printed proceedings.

Dr. Hamilton, of Palestine, tendered his resignation as Secretary, urging ill-health as the cause, when, upon motion of Dr. Chambers, the consideration of the subject was postponed until the afternoon session.

Dr. H. R. Payne, of Marshall, moved that the calling the roll of members be deferred until the afternoon session. Carried.

The report of the Treasurer was in order, but without response from that officer.

Dr. F. R. Payne proposed the name of Dr. V. R. Bridges, of Salisbury, in Coles County, for membership; referred to Board of Censors.

Dr. Chambers moved that a committee of three be appointed by the chair, to report upon the best mode of having the proceedings, together with the reports made to this society, published; and that the committee report to-morrow morning. Carried. And Drs. Chambers, F. R. Payne, and Gorham were appointed.

EVENING SESSION.

The Vice-President, Dr. D. W. Stormant, in the chair.

On motion, the resignation of Dr. C. M. Hamilton, as Secretary, was received, whereupon the President appointed Dr. W. M. Chambers Secretary *pro tem.;* and, on motion, Dr. F. R. Payne was appointed Assistant Secretary.

Dr. F. R. Payne moved that the name of Dr. Charles Gorham, which has, by some strange mistake, been heretofore omitted by the Secretary, be now inserted as a member. Carried.

The roll of members was then called, and the following gentlemen were found to be present, viz.:—Drs. E. C. Banks, of Charleston; C. M. Hamilton and J. M. Logan, of Palestine; C. Johnson, D. O. McCord, C. Gorham, and William Leachman, of York; F. R. Payne, H. R. Payne, and L. C. Churchill, of Marshall; D. W. Stormant, and J. M. Steele, of Grandview; J. D. Mitchell, of Darwin; W. R. Duffield, of Sullivan; and Wm. M. Chambers, of Charleston.

Dr. C. M. Hamilton was, on motion of H. R. Payne, appointed Treasurer *pro tem.*

Dr. D. O. McCord, of York, read an interesting paper on the Theory of Intermittent Fever, discussing the subject at length, in a forcible and logical manner.

Dr. Banks, Chairman of the Board of Censors, reported favorably on the petitions of Dr. Bridges and Daniel Bowers, M.D.

The vote was then taken upon the reception of Dr. Bowers as a member—unanimous in his favor. Dr. Bridges, on motion, was requested to read his Thesis to-morrow morning.

The discussion of Dr. McCord's paper was declared in order. Dr. Chambers commended the report, and hoped the writer would be appointed to continue the subject.

Dr. H. R. Payne read a paper upon Puerperal Convulsions, in which he gave the whole subject a thorough investigation, and reported two or three very interesting cases. The paper was lengthily discussed by Drs. Banks, Chambers, Mitchell, H. R. Payne, McCord, Churchill (who reported some cases he had recently treated, which gave additional interest to the subject,) F. R. Payne, and Johnson. [Such observations, and reports, and discussions, are what go to make up the sum of our professional knowledge—*Sec't.*]

Dr. Chambers reported two cases of Typhoid Pneumonia, which were discussed by Drs. H. R. Payne, Johnson, McCord, Mitchell, Banks, Steel, Stormant, F. R. Payne, Logan, and Chambers.

Dr. Johnson reported two cases of Pneumonia, which were discussed by Drs. Chambers and Johnson.

NIGHT SESSION.

Dr. Stormant in the chair.

Dr. Chambers read his essay upon "Substitutes for Quinine," in the conclusion of which he offered a series of resolutions, the purport of which was, to appoint a committee and continue the investigation of the subject by having tabular information from the members, the Secretary to furnish tables to the members. Discussion of paper and resolutions postponed until to-morrow.

Dr. F. R. Payne then read an entertaining paper on "Practical Medicine," giving its history in a complete and finished manner, which did great credit to his powers of research, as well as his practical application of things.

On motion, Dr. Banks is to report the proceedings of the American Medical Association, at 10 o'clock to-morrow morning.

On motion of Dr. Banks, each member was allowed six copies of the Constitution and By-Laws of the Society recently published.

Adjourned to meet to-morrow morning at 8 o'clock.

THURSDAY MORNING, May 29th, 1856.

The Society met pursuant to adjournment, Dr. Stormant in the chair. Minutes of proceedings yesterday, read and adopted.

Dr. Logan offered a resolution, making it obligatory upon the members to sign the Constitution. Dr. Chambers moved to amend by appointing a committee who should ascertain the names and address of all the members of the Society, or devise some plan by which the Secretary could get in possession of such information. Amendment added, and Drs. Banks, F. R. Payne, and Gorham were appointed the committee, with instructions to report in an hour.

Dr. Bridges, a candidate for membership, read a paper, reporting a case as a substitute for a Thesis, which, on motion of H. R. Payne, was received, whereupon the candidate was balloted for and unanimously elected a member, and took his seat with the meeting.

Dr. Charles Johnson presented a report from Dr. S. Thompson, of Albion, on Recto-Vaginal Fistula, which was read by the Secretary. Discussed by Dr. Logan, of Palestine.

Dr. Logan offered the following:—

*Resolved*, That Drs. Hinkle and Helm, of Carlisle, Ia., (who are present,) be requested to participate in the proceedings of the Society. Adopted.

Dr. Chambers, from the committee on the best means of securing the publication of the transactions of this Society, read the following report:—

Inasmuch as the medical profession are the guardians of the public health, as well in preventing as is curing disease, and, as a consequence, the public are deeply interested in the transactions of this Society; inasmuch as its members are now engaged in the investigation of the causes, prevention, and best mode of curing the diseases peculiar to this State: Therefore,

*Resolved*, That a committee be appointed by this Society, consisting of one member from each county represented therein, whose duty it shall be to prepare a memorial upon the subject, and transmit a copy thereof to the Secretary, who shall publish the same in circular form; and, upon the election of the members of the next Legislature of the State, shall transmit a copy thereof to each Senator and Representative, and then, on the assembling of said Legislature, the committee shall urge upon that body to make an appropriation sufficient to defray the publication of the practical facts and transactions of this Society annually.

W. M. Chambers.
F. R. Payne.
C. Gorham.

The report was discussed at great length by Drs. Banks, McCord, Mitchell, Logan, H. R. Payne, and Chambers.

Dr. Banks, chairman of committee on the resolution offered by Dr. Logan as afterwards amended, reported that the whole subject of membership and residence of members, be referred to Dr. Hamilton former Secretary. Report, on motion, referred back to committee, with further time.

Dr. F. R. Payne offered a resolution upon the subject of publication. Laid on table.

Dr. H. R. Payne offered a resolution which did not come into hands of Secretary. Dr. McCord also offered one which he did not get—both of which were tabled.

Dr. Logan, Chairman of the Standing Committee on Indigenous Botany, (appointed at last meeting,) tendered his resignation of the position, (cause ill-health,) and recommended the appointment of Dr. Churchill, of Marshall, in his stead. Resignation accepted, and appointment of Dr. Churchill confirmed.

Dr. Banks made a report of the proceedings of the American Medical Association at its last meeting, which he had attended as a delegate from this Society.

Dr. Chambers' Essay on Substitutes for Quinine was brought up for discussion; and, on motion, the preamble and resolutions appended thereto were adopted, and Drs. S. Thompson, Bridges, and Bowers were appointed the committee to carry out the object of the resolution.

The President declared the selection of a place for next meeting in order, when, upon motion, Grandview was unanimously selected.

Dr. Chambers moved that a committee be appointed to select special subjects to be reported on at the annual meeting. Carried; and Drs. F. R. Payne, Hamilton, and McCord were appointed.

The President appointed Dr. H. R. Payne, of Marshall, to deliver the annual address.

The Secretary, on motion of Dr. Logan, read the delinquent list; and, on motion, the resolution of Dr. York, adopted at the last meeting, in regard to the expulsion of members for non-attendance and unpaid dues, was rescinded; and, further, that Dr. Hamilton be directed to open a correspondence with Drs. Duncan and Washburn, former Treasurers, and report at next meeting the exact state of our finances as far as possible.

Dr. Banks, Chairman of Board of Censors, reported favorably on the petition of Dr. Hinkle, and, upon ballot, he was unanimously elected.

Dr. F. R. Payne, Chairman of Committee on Special Subjects, to be reported on at next meeting, announced the following, and upon the adoption thereof the President appointed the gentlemen whose names are opposite the subjects to report thereon, viz.:—

Milk Sickness, Dr. J. M. Logan.

Scrofula, Dr. T. D. Washburn.

Pneumonia, Dr. H. N. Davis.

Prolapsus Uteri, Dr. C. M. Hamilton.

Cholera Infantum, Dr. D. W. Stormant.

Veratrum Viride, Dr. C. Gorham.

Causes Intermittent Fever, Dr. V. R. Bridges.

Epidemic Dysentery, Dr. D. Bowers.

Acute Bronchitis, Dr. T. D. Mitchell.

Diagnosis in Typhoid and Typhus Fever, Dr. D. O. McCord.

Use and Abuse of Alcohol, Physiologically Considered, Dr. J. M. Hinkle.

Stomatitis Materna, Dr. J. M. Steele.

Dr. Chambers offered the following:—

*Resolved*, That the sincere thanks of the Society are gratefully tendered the acting President, Dr. Stormant, for the calm, dignified, and courteous manner in which he has conducted the proceedings of the present session. Carried by a rising vote unanimously.

Dr. Mitchell, of Darwin, offered the following, viz.:—

*Resolved*, That physicians applying for membership to this Society, who are not graduates of a regular college, shall be required to present to the Censors satisfactory evidence of their standing, as physicians, in the regular profession; and such evidence shall take the place of an examination on the different branches of medicine, and as a thesis. Lays over until annual meeting.

Dr. Chambers, of Charleston, offered the following resolution:

*Resolved*, That the thanks of the Society are due the proprietors of this house, for the use thereof during the present meeting, and are hereby most cordially tendered. Carried.

It was moved, that a copy of the proceedings of this meeting be furnished the *North-Western Medical and Surgical Journal*, and the various papers of Marshall, Charleston, Paris, Palestine, and the *Vincennes Gazette*, with the request that they publish.

Adjourned to meet at Grandview, on the 4th Wednesday in October. D. W. STORMANT, *President*.

WM. M. CHAMBERS, *Sec. P. T.*

